# Predicting serum phosphate levels in very preterm infants using machine learning

**DOI:** 10.3389/fped.2026.1881474

**Published:** 2026-07-15

**Authors:** Åsbjørn S. Westvik, Oliver Tomic, Charlotte Tscherning, Sissel J. Moltu

**Affiliations:** 1 Institute of Clinical Medicine, University of Oslo, Oslo, Norway; 2 Department of Neonatal Intensive Care, Oslo University Hospital, Oslo, Norway; 3 Faculty of Science and Technology, Norwegian University of Life Sciences, Ås, Norway

**Keywords:** hypophosphatemia, machine learning, prediction model, preterm infant, refeeding syndrome

## Abstract

**Background:**

Hypophosphatemia in very preterm and small for gestational age (SGA) infants is common and associated with serious complications. Diagnosis relies on phosphate concentrations, measured in serum or plasma. Machine learning (ML) may enable indirect estimation of serum phosphate based on routinely collected clinical data and results from blood gas analysis.

**Objectives:**

To (1) describe first week electrolyte trajectories in the ImNuT-trial (Clinicaltrials.gov ID: NCT03555019), and (2) develop and externally validate ML models to estimate serum phosphate levels during the first postnatal week in very preterm infants, using routinely collected clinical and nutritional data.

**Methods:**

We performed a retrospective analysis of 120 infants born <29 weeks' gestation, all managed under a standardized nutritional protocol. Electrolyte trajectories (calcium, potassium, sodium, phosphate) were described using daily means stratified by hypophosphatemia and SGA status. For ML, we defined three regression tasks: concurrent phosphate prediction at blood gas analysis, and forecasts 12 h and 24 h ahead. Twenty-two candidate predictors from blood gases, anthropometry, SGA status and nutrient intakes were subjected to RENT-based variable selection within leave-one-group-out cross-validation. Multiple algorithms (elastic net, gradient boosting, random forests, k-nearest neighbours, kernel ridge, XGBoost) were trained under three preprocessing strategies (complete cases, complete cases without outliers, imputed data). Performance was evaluated with RMSE and *R*^2^ using nested cross-validation and an independent external cohort of 40 very preterm infants.

**Results:**

Among 119 infants with phosphate measurements (607 samples), 33.6% developed hypophosphatemia in week one. SGA infants had a markedly higher and earlier risk of hypophosphatemia compared to appropriate-for-gestational-age infants (log-rank *p* < 0.0001). Six predictors (calcium, potassium, gestational age, SGA status, birth weight *z*-score, daily weight *z*-score) were consistently retained across all prediction tasks. Elastic net models performed best externally on all three prediction tasks. Performance was robust across preprocessing strategies.

**Conclusions:**

SGA status is a major determinant of early hypophosphatemia risk in very preterm infants. ML models using routinely available clinical and nutritional data can moderately but consistently estimate serum phosphate levels, including 12–24 h ahead, and may serve as screening tools to guide nutritional management and reduce unnecessary blood sampling. Prospective validation is warranted before clinical implementation.

## Introduction

Hypophosphatemia in preterm infants is associated with severe complications including sepsis (1), chronic lung disease (2), intraventricular hemorrhage (3), neurodisability and death (4). The incidence of hypophosphatemia in preterm infants is high, ranging from 20% to 90%, with the wide variation attributed to differing definitions, gestational age, and nutritional strategies (5). All very preterm (born before 32 weeks) and especially extremely preterm infants (born before 28 weeks) are at increased risk of hypophosphatemia because they have high phosphate demands due to rapid postnatal growth and limited phosphate supply, particularly if dependent on parenteral nutrition (6, 7). Infants who are both preterm and small for gestational age (SGA), defined as birthweight below the 10th centile (8), and due to intra uterine growth restriction have an additional risk to be phosphate depleted at birth. Thus, both prematurity itself and SGA status contribute to the overall vulnerability to hypophosphatemia, through partly overlapping but distinct mechanism.

Nutritional strategies, which typically consist of a combination of parenteral and enteral nutrition during the first postnatal week, must balance macro- and micronutrient provision to achieve adequate growth while limiting the risk of adverse events (9). For instance, regimens with high protein intakes require correspondingly high phosphate supplies (1, 3, 10), but phosphate supplementation in parenteral solutions is often limited by compatibility issues. On the other hand, enteral supplementation, albeit used, may pose a risk due to the high osmolarity of the products (11, 12). We have previously demonstrated that very preterm infants are at risk of hypophosphatemia and neonatal refeeding syndrome when exposed to an unbalanced phosphate to protein ratio (1). In response, nutritional practice at our center was modified. In a more recent study from our center, the ImNuT-trial, all infants followed a standardized nutritional protocol to accommodate present recommendations for nutrient intakes in very preterm infants (9, 13–15). Despite optimizing nutrition in very preterm infants has been shown to reduce the prevalence and duration of hypophosphatemia (16), hypophosphatemia remains a concern in this population.

Diagnosis of hypophosphatemia is established through laboratory measurements of serum or plasma phosphate, with definitions ranging from <0.8 mmol/L to <1.6 mmol/L (5). Each serum sample requires 125–250 μl of blood, which represents a significant amount for a preterm infant. Frequent blood sampling contributes to iatrogenic anemia in this population, often necessitating blood transfusions (17, 18). Skin punctures also cause stress and pain (19, 20), which in turn is associated with altered brain development (21). Methods to both limit the number of and the volume of blood samples are desirable.

Machine learning (ML) is a branch of computer science that uses algorithms to identify patterns in data and make predictions based on those patterns (22). ML has become an increasingly important research tool in medicine, where it can analyze large and complex datasets and incorporate far more variables simultaneously than a human can reasonably process (23). Recent studies highlight the growing application of machine learning approaches in neonatal and perinatal medicine (24–27) underscoring the increasing interest in leveraging data-driven methods in this field. Building on this evolving landscape, the present study focuses specifically on predicting serum phosphate levels in very preterm infants, addressing the need for more knowledge in metabolic management in this patient group.

Studies provide strong evidence of an inverse relationship between phosphate and calcium concentrations in blood, and a positive relationship between phosphate and potassium concentrations (1, 3, 28). Very preterm infants are frequently monitored with blood gas analysis (BGA), a near-patient method that also provides calcium and potassium values and only requires 25 μl of blood per sample. Based on these established relationships between phosphate, calcium, and potassium in the first postnatal week in preterm infants, we hypothesize that serum phosphate levels can be estimated indirectly from routinely available clinical and nutritional data using ML models. Integrating information from BGA, nutritional intake, and patient-specific characteristics (chronological age, gestational age, weight, and SGA status) may allow prediction of serum phosphate levels, both concurrently with the BGA and ahead of time. Our study has two aims. The first is to describe electrolyte trajectories during the first postnatal week in the ImNuT-cohort, with particular emphasis on SGA status and hypophosphatemia. The other aim is to develop and evaluate ML models to estimate serum phosphate levels during the first postnatal week in very preterm infants from the ImNuT cohort, using routinely collected clinical and nutritional data. If the ML models prove sufficiently accurate, they could be incorporated into a clinical decision support system to optimize nutritional management in very preterm infants, prevent hypophosphatemia, facilitate timely initiation of treatment, and reduce the need for additional blood sampling.

## Methods

### Study design and population

Our retrospective clinical dataset was acquired from the ImNuT-trial (NCT03555019). The trial cohort included 120 very preterm infants born before gestational age 29 + 0, recruited from April 2018–January 2021 at Oslo University Hospital, Norway. Ethical approval (2017/1067) was given to develop machine learning models. The demographics of the ImNuT-trial has been published earlier (29). Nutritional management and phosphate supplementation were standardized. The nutritional protocol has previously been published (13), and it includes a standardized setup of enteral and parenteral nutrition for the first 10 days of life. The phosphate intake was on average 40.0 (range 18.7–66.0) mg/kg/d in week one. Hypophosphatemia was defined as serum phosphate <1.4 mmol/L (1, 3). SGA was defined as having birth weight lower than the 10th centile. Weight *z*-score expressed an infant's weight relative to a reference population as the number of standard deviations from the reference mean, where the ImNuT-study used the Niklasson growth chart for preterm infants as the reference (30). Inclusion criteria for the ImNuT-trial were: infants born at Oslo University Hospital with GA <29 weeks, incusion before 48 h of age and signed informed consent. Exclusion criteria were major congenital malformations, chromosomal abnormalities and other genetic diseases diagnosed prenatally or detected during the study period, and critical illness with short life expectancy (13).

### Data collection and electrolyte analyses

Throughout this manuscript, N refers to the number of unique individuals and *n* to the total number of observations within the relevant dataset. Data was restricted to the first week of life. Of the *N* = 120 infants, a total of *n* = 1,960 BGAs were included in the analysis, each infant contributing multiple measurements. The number of observations per infant varied, ranging from 2 to 42, with a median of 16. In addition to BGAs, daily weight *z*-scores and nutritional intake were collected.

Electrolyte trajectories during the first week of life were analyzed for calcium, potassium, sodium and phosphate. Infants were classified as having hypophosphatemia if at least one serum phosphate measurement during the first week of life met this criterion. Trajectories were visualized as daily means with standard deviations, presented separately for infants with and without hypophosphatemia. All electrolyte variables in this study were obtained from the BGA, except from phosphate which was obtained from serum.

### Machine learning tasks

For the prediction of serum phosphate levels, we defined three distinct prediction tasks, based on the timing of the target phosphate measurement relative to a reference blood gas analysis (BGA) event:

1. Concurrent prediction (T0): Predicting serum phosphate level concurrently with a BGA.
2. 12-Hours Ahead Prediction (T12): Forecasting serum phosphate levels 12 h after a reference BGA.
3. 24-Hours Ahead Prediction (T24): Forecasting serum phosphate levels 24 h after a reference BGA.

Previous phosphate measurements were not included as predictors in order to enable indirect estimation based solely on routinely available clinical and biochemical data at each time point.

For clarity and consistent reference throughout this manuscript, datasets, flowcharts and models associated with these three prediction tasks will be denoted by the prefixes T0, T12, and T24, respectively. In addition, the flowcharts use consistent coloring (T0 = red, T12 = green and T24 = blue).

### Datasets

To maintain clarity for clinical readership, we use the terms “variables” and “response variable” rather than the ML nomenclature “features” and “target”. In total, 23 variables were collected for data exploration and model development. Twenty-two of the variables were used as potential input parameters to the models (Table 1), whereas phosphate-level was used as the response variable in the ML regression models.

**Table 1:** Candidate input variables for the ML regression models.

Available input variables from ImNuT	T0	T12	T24
pH			X
Bicarbonate			
Glucose			X
Calcium (Ca)	X	X	X
Potassium (K)	X	X	X
Sodium (Na)	X	X	
Age at sampling		X	X
Base excess			
Chloride (Cl)			
Lactate			
O2	X		
pCO2			
Bilirubin			
Haemoglobin (Hb)	X		X
Gestational age	X	X	X
Birth weight *z*-score	X	X	X
Daily weight *z*-score	X	X	X
SGA-status	X	X	X
Protein g/kg/d	X		X
Fluid mL/kg/d			
Phosphorus mg/kg/d			
Calcium mg/kg/d			

The final three columns show retained variables after variable selection for each prediction task (T0, T12, and T24).

For external validation, we used an anonymous dataset of preterm infants (*N* = 40).

### Data preprocessing

#### Variable selection

**Figure 1:**
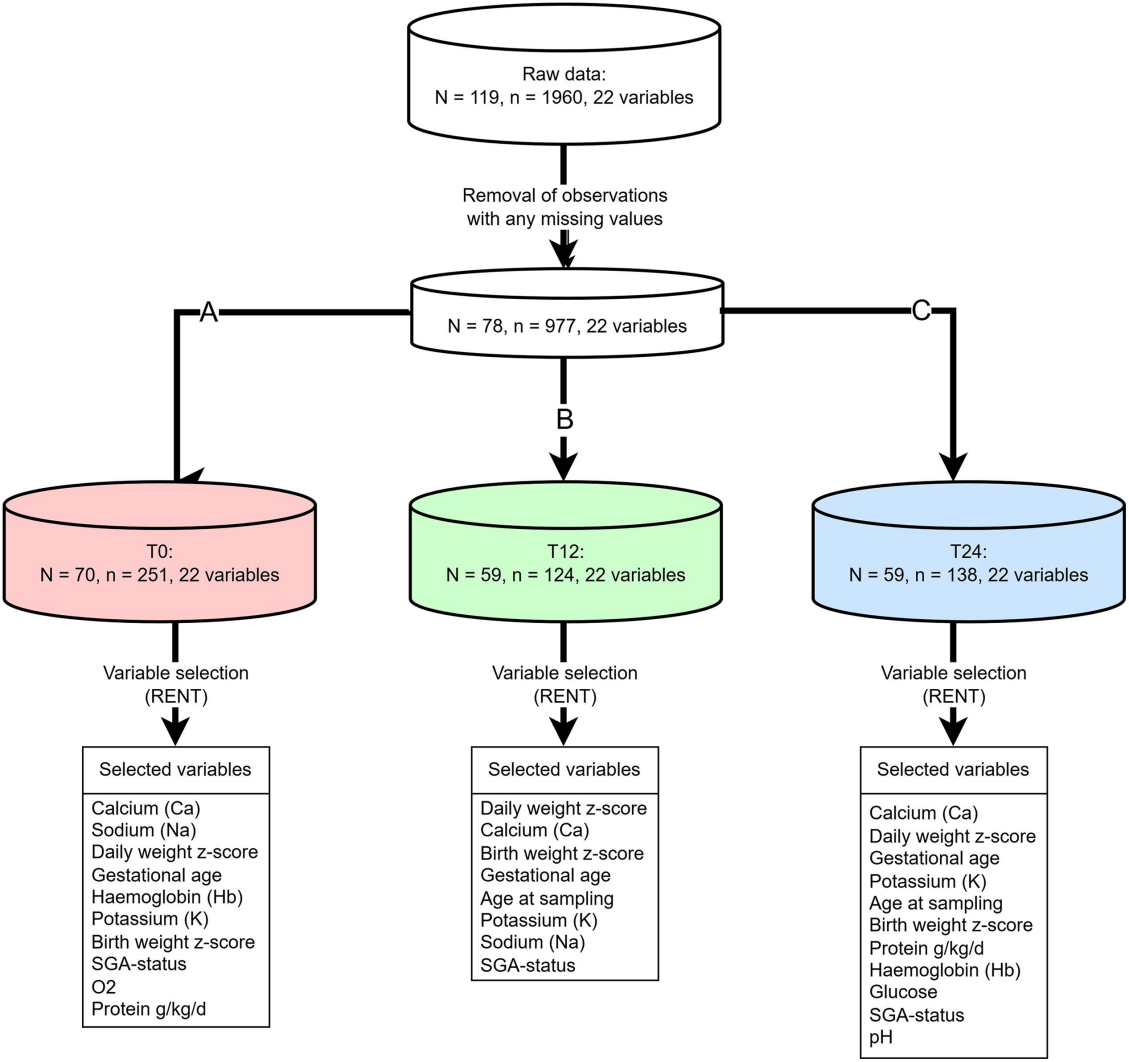
Flowchart, dataset creation for variable selection. Selected variables are listed in descending order by selection frequency across runs. **(A)** Observations matched to phosphate value taken within 1 h of BGA. **(B)** Observations matched to phosphate value taken 12 h after BGA. **(C)** Observations matched to phosphate value taken 24 h after BGA.

Distinct datasets were created for each prediction problem (T0, T12 and T24) as shown in Figure 1. Variable selection was performed using RENT (repeated elastic net technique) (31) embedded within a leave-one-group-out cross-validation (LOGOCV) framework, where each group represented all measurements from a single patient. This approach allowed the variable selection process to be repeated across multiple resampled training sets.

Across these repeated runs, the frequency with which each variable was selected was calculated. Variables selected in more than one third of the repeated runs (>33%) were retained for model development. This threshold was chosen to limit the number of predictors and exclude unstable noisy variables. Table 1 and Figure 1 present the set of variables selected for model development across the three prediction tasks, where we created separate datasets for each task. For the T12 datasets, observations were matched to phosphate values obtained 10–14 h after BGA to approximate a 12-h time point while allowing for routine variation in sampling times. For the T24 datasets we matched observations to phosphate values obtained 22–26 h after BGA to approximate a 24-h time point.

#### Missing values, imputation and outliers

**Figure 2:**
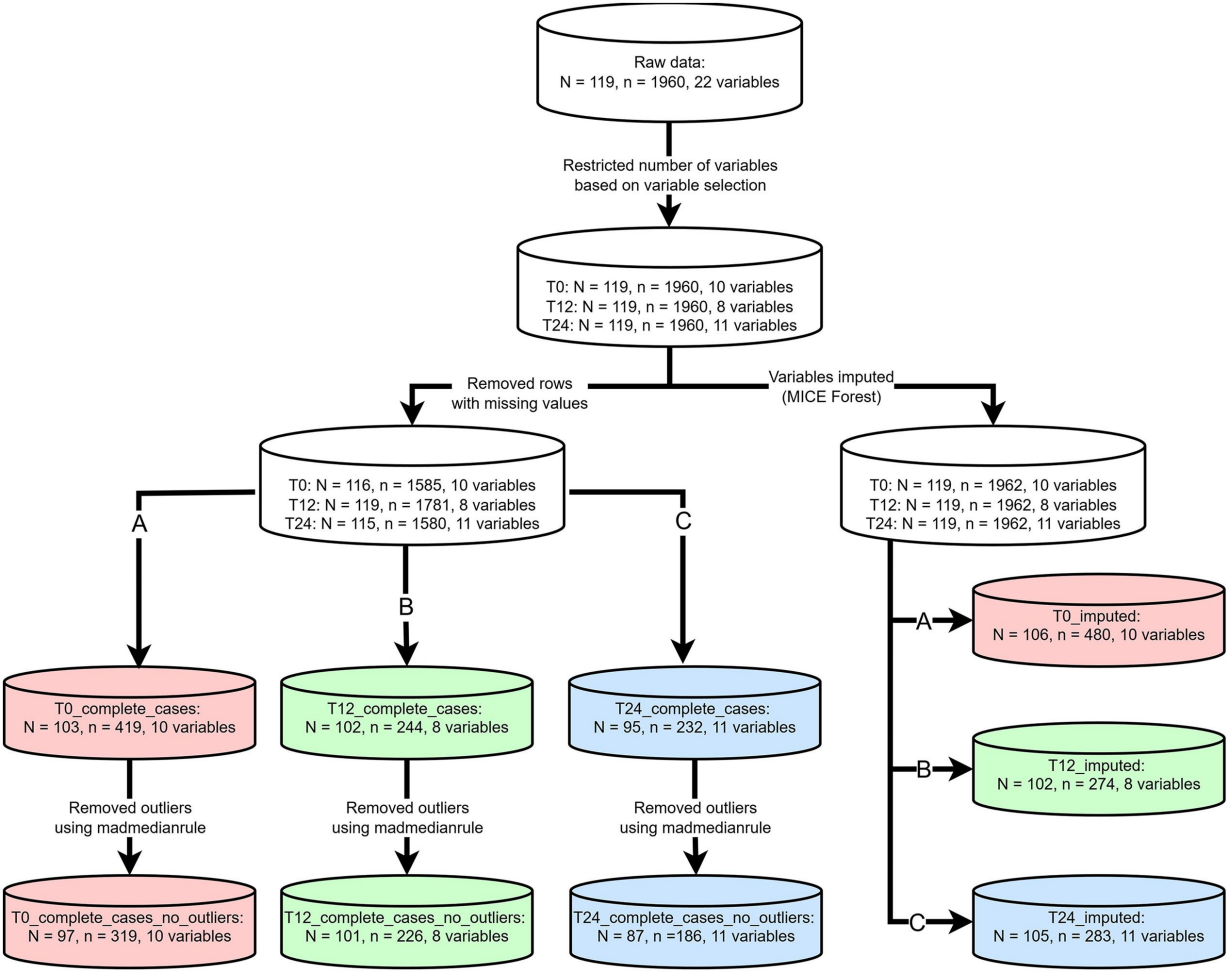
Flowchart of datasets used in model development. **(A)** Observations matched to phosphate value taken within 1 h of BGA. **(B)** Observations matched to phosphate value taken 12 h after BGA. **(C)** Observations matched to phosphate value taken 24 h after BGA.

Certain variables on the BGA had frequent missing values, and some observations contained extreme outliers. Because the ML algorithms evaluated require complete input data, missing data were addressed using three approaches: 1) complete case analysis, excluding observations with any missing values, 2) complete case analysis with additional exclusion of outliers, and 3) imputation of missing values, with no outlier exclusion. This strategy allowed us to evaluate the robustness of our ML models by examining how variations in data preprocessing affected model performance. By comparing results across these three approaches, we could assess the extent to which data integrity influenced predictive accuracy and determine whether the models yielded consistent insights despite changes in the input data. Figure 2 illustrates the workflow based on the three data pre-processing approaches mentioned above. Of note, after variable selection the rows without any missing values increased since the excluded variables often contained missing data. For outlier removal we used the median absolute deviation (MAD) median rule (32). Imputation was performed using the Python package MICEforest (33).

**Figure 3:**
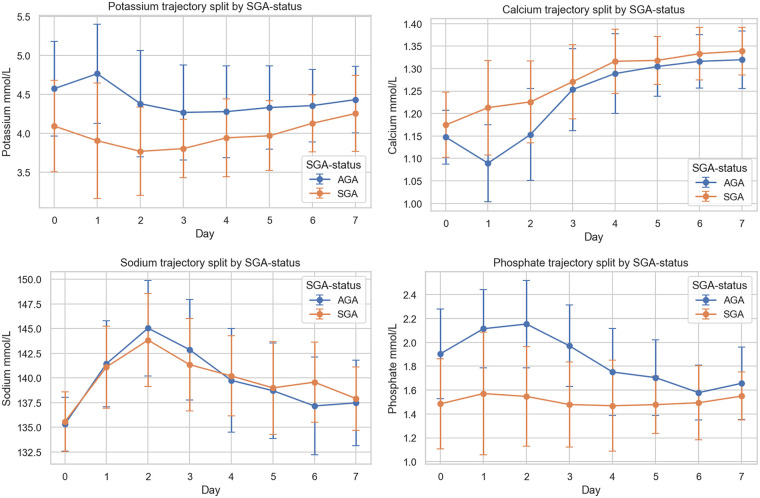
Electrolyte trajectories for week one, split by SGA-status, where AGA are infants born appropriate for gestational age.

### Machine learning model development

A selection of machine learning regression algorithms was applied to the data: elastic net, gradient boosting (GBR), random forests (RF), k-nearest neighbors (KNN), kernel ridge (KR) and XGBoost. These algorithms were chosen to represent a broad range of approaches with focus on predictive performance over interpretability. Several of these methods (GBR, RF, KNN, KR and XGBoost) are capable of capturing nonlinear relationships and interactions between variables without requiring them to be specified in advance, allowing for a data-driven exploration of complex predictor dependencies in relation to serum phosphate levels. In contrast, elastic net models assume additive relationships unless interaction terms are explicitely defined. For algorithms requiring scaled inputs, preprocessing was performed using either standard scaling (SS) or the Yeo-Johnson (YJ) power transformation. The Yeo-Johnson transformation reduces skewness and approximates Gaussian variable distributions. Optimal hyperparameters of the models were determined using the Optuna (34) framework. Model performance was then evaluated separately for each preprocessing approach, using nested LOGOCV, with a group defined as the sequential data from one single patient. The models' predictive performance was measured with the performance metrics root mean squared error (RMSE) and R^2^.

### Anonymous data and external validation

External validation was performed using an independent, anonymized dataset of 40 very preterm infants. Separate datasets (ext_T0, ext_T12 and ext_T24) were created for each prediction task (T0, T12, and T24). The number of observations differed across tasks (ext_T0: *n* = 43 observations; ext_T12: *n* = 20 observations; ext_T24: *n* = 20 observations). All required input variables were available in the external dataset, apart from oxygen saturation (O2) which was included as a predictor for the T0 task. Of note, the weight *z*-scores of the external dataset were obtained from the Skjærven growth chart (35). Consequently, model hyperparameters for the T0 task were re-optimized on the internal dataset using the reduced variable set prior to external evaluation. For the T12 and T24 tasks, no additional hyperparameter optimization was required, as all variables were available. Predictive model performance was also evaluated with RMSE and R^2^. In a secondary analysis, predicted phosphate values were dichotomized using the clinically relevant threshold of <1.4 mmol/L to evaluate classification performance. Sensitivity, specificity and negative predictive values were calculated.

A comparison of the demographic differences between the ImNuT-cohort and the cohort in the external validation dataset are presented in Table 2.

**Table 2:** Demographic differences between imNuT-cohort and the external validation cohort.

Characteristic	ImNuT-cohort	External validation dataset
Infants	120	40
Gestational age (mean)	26^+6^	28^+2^
Birth weight (mean)	798 g	1,013 g
Phosphate values in mmol/L (range)	0.6–3.05	0.18–2.5
Percentage SGA	24%	33%

### Software

For the data preparation the R Statistical Software (36, 37) was used, and for modelling we used the Python package scikit-learn (38). Figures were made in both R (39) and in Python (40, 41).

## Results

### Electrolytes

Figure 3 shows the electrolyte trajectories of potassium, sodium, calcium and phosphate in the ImNuT-cohort. Data points show group means; error bars indicate ±1 standard deviation

#### Phosphate

In total, 119/120 of the ImNuT-trial infants had at least one phosphate measurement (*n* = 607, µ = 1.81 mmol/L) in week one. 40 patients (33.6%) had hypophosphatemia (phosphate value <1.4 mmol/L) in the same week.

The Q-Q plot of the phosphate value (Supplementary Figure S1) showed an approximately normal distribution with minor tail deviations.

### Data exploration

Initial data exploration assessed pairwise linear correlation between each input variable and the phosphate concentrations using the Pearson correlation coefficient (r) (Table 3). For SGA-status, Spearman rank correlation coefficient was used instead, as the binary nature of the variable violates the assumptions of Pearson correlation coefficient.

**Table 3:** Pearson correlation coefficients between each input variable and the phosphate concentration response variable.

Variable	T0	T12	T24
pH	0.07 (0.24)	0.12 (0.17)	0.23 (0.01)
Bicarbonate	−0.04 (0.49)	0.07 (0.43)	0.13 (0.12)
Glucose	0.04 (0.48)	0.07 (0.45)	0.12 (0.18)
Calcium (Ca)	−0.59 (<0.01)	−0.53 (<0.01)	−0.57 (<0.01)
Potassium (K)	0.35 (<0.01)	0.42 (<0.01)	0.42 (<0.01)
Sodium (Na)	0.36 (<0.01)	0.06 (0.54)	0.08 (0.33)
Age at sampling	−0.42 (<0.01)	−0.31 (<0.01)	−0.42 (<0.01)
Base excess	−0.03 (0.69)	0.10 (0.28)	0.18 (0.03)
Chloride (Cl)	0.29 (<0.01)	−0.01 (0.89)	0.02 (0.81)
Lactate	0.08 (0.21)	0.11 (0.23)	0.05 (0.59)
O2	−0.07 (0.27)	0.13 (0.16)	<0.01 (0.97)
pCO2	−0.09 (0.13)	−0.08 (0.4)	−0.11 (0.21)
Bilirubin	−0.06 (0.31)	−0.28 (<0.01)	−0.25 (<0.01)
Haemoglobin	0.14 (0.02)	−0.08 (0.41)	0.16 (0.07)
Gestational Age	−0.26 (<0.01)	−0.39 (<0.01)	−0.31 (<0.01)
Birth weight *z*-score	0.42 (<0.01)	0.54 (<0.01)	0.41 (<0.01)
Daily weight *z*-score	0.48 (<0.01)	0.60 (<0.01)	0.54 (<0.01)
SGA-status*	−0.38 (<0.01)	−0.42 (<0.01)	−0.34 (<0.01)
Protein g/kg/d	−0.32 (<0.01)	−0.33 (<0.01)	−0.45 (<0.01)
Fluid ml/kg/d	−0.26 (<0.01)	−0.17 (0.06)	−0.32 (<0.01)
Phosphorus mg/kg/d	−0.39 (<0.01)	−0.38 (<0.01)	−0.39 (<0.01)
Calcium mg/kg/d	−0.30 (<0.01)	−0.29 (<0.01)	−0.32 (<0.01)

For each correlation coefficient, the corresponding *p*-value is in parentheses.

* Spearman correlation coefficient used for SGA-status.

**Figure 4:**
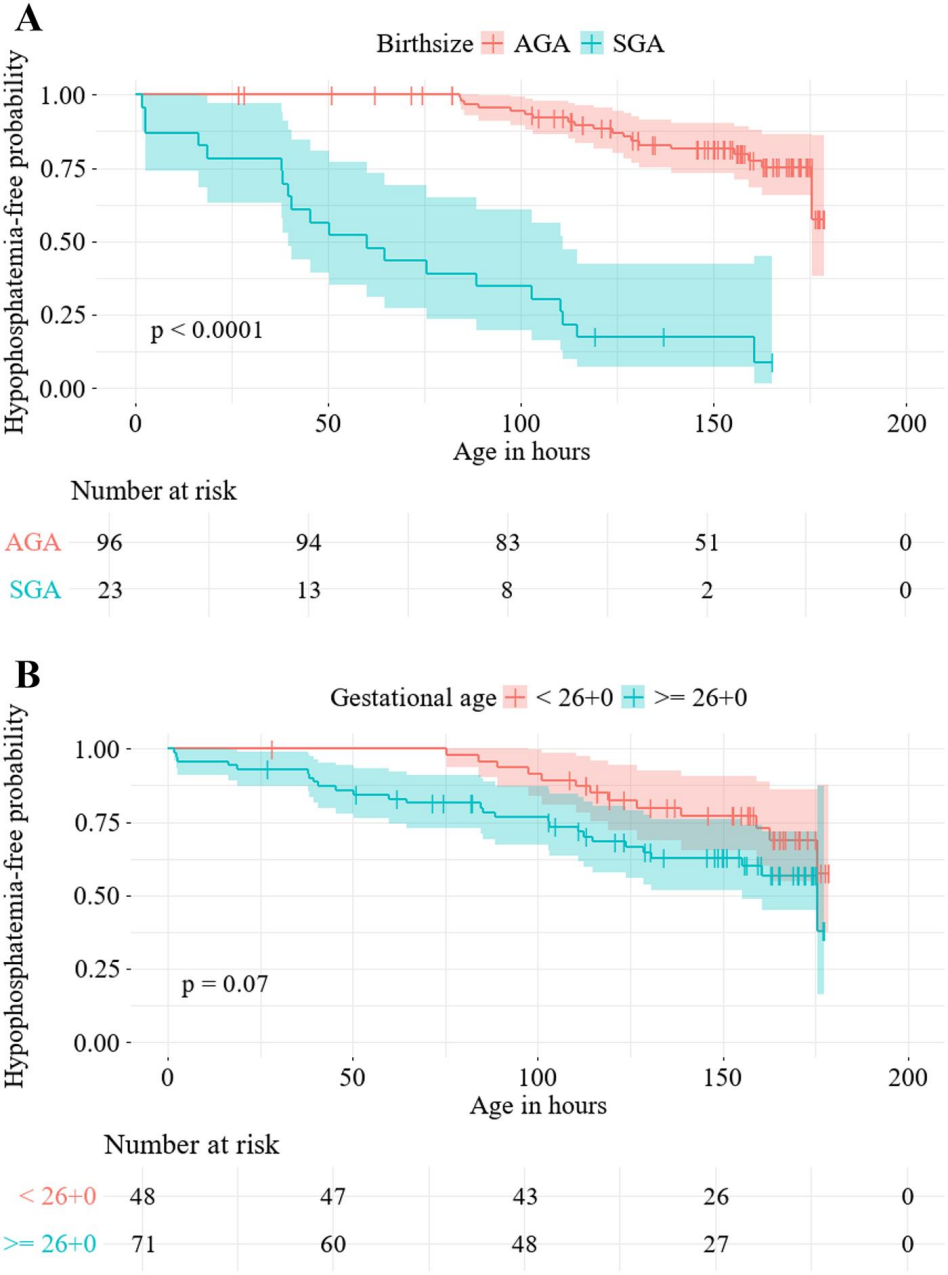
Kaplan–Meier plots showing first event of hypophosphatemia. Colored bands represent the 95% confidence interval. **(A)** Infants born appropriate for gestational age (AGA) vs. SGA. **(B)** Patients born immature (GA < 26 weeks) vs. less immature (GA ≥ 26 weeks).

We explored the onset of hypophosphatemia in our data with respect to both SGA-status and gestational age using Kaplan–Meier plots (Figure 4).

As can be seen in Figure 4, the risk of hypophosphatemia during the 1st week of life was significantly greater (*p* < 0.0001) for infants born SGA compared to infants born appropriate for gestational age (AGA). In the SGA group, several infants developed hypophosphatemia within the first few hours after birth, and the hypophosphatemia-free survival probability declined rapidly, reaching approximately 0.2 by around 120 h of age. In contrast, in the AGA group, the earliest hypophosphatemia events occurred much later, at approximately 80 h of age, and the decline in hypophosphatemia-free survival was markedly slower.

When comparing the curves of infants GA < 26 weeks with those GA ≥ 26 weeks one can see the GA ≥ 26 infants show both earlier and more frequent hypophosphatemia events. However, the difference between the two groups is smaller and borderline statistically non-significant (*p* = 0.07).

We examined the association between GA and SGA status using logistic regression, modeling GA both as a continuous variable and as a binary predictor (<26 weeks vs. ≥26 weeks). As a continuous predictor, each additional week of gestation was associated with 34% higher odds of being SGA (OR = 1.34, 95% CI = 1.01–1.83, *p* = 0.053). As a binary predictor, GA ≥ 26 weeks was associated with higher odds of being SGA compared to those born before 26 weeks of gestation (OR = 4.23, 95% CI = 1.47–15.4), confirmed by Fisher's exact test (*p* = 0.010).

### Model performance

All ML algorithms were calibrated and tested on both internal and external datasets (Supplementary Table 1). Elastic nets performed best on the external datasets, and the results from both internal and external validation are listed in Table 4. Results when using the best performing models as a classifier are shown in Table 5.

**Table 4:** Scores of the best performing models (on external datasets) for different prediction tasks.

Best performing model	Dataset	RMSE	R^2^
Elastic net SS	T0	0.297	0.469
Elastic net SS	T12	0.290	0.526
Elastic net YJ	T24	0.297	0.519
Elastic net SS	extT0	0.308	0.562
Elastic net SS	extT12	0.360	0.581
Elastic net YJ	extT24	0.513	0.265

A lower RMSE, expressed in mmol/L, and a higher R^2^ indicates better performance.

**Table 5:** Results using the best performing models as a classifier on the external dataset, with hypophosphatemia defined as phosphate <1.4 mmol/L.

Performance metric	extT0	extT12	extT24
Sensitivity	0.83	0.93	0.92
Specificity	0.38	0.67	0.43
Negative predictive value	0.50	0.80	0.75

Although not shown here, the model performance was similar when the models were calibrated and tested on datasets without outliers and datasets with imputed missing values (Supplementary Table S2).

**Figure 5:**
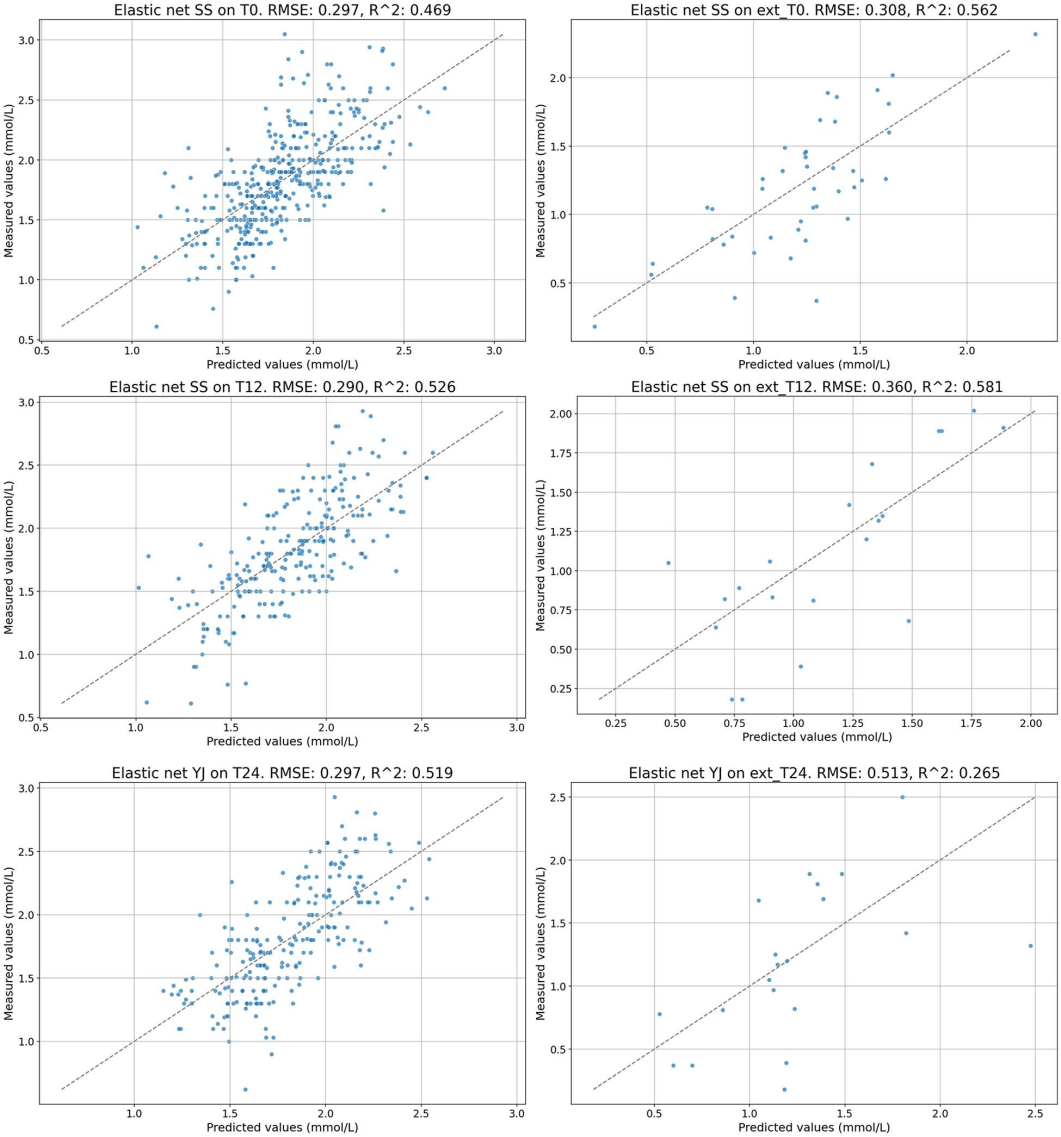
Scatter plots of predicted value vs. actual value for the best performing models on internal and external validation.

Figure 5 shows the scatter plots of predicted vs. actual values in the best performing models.

## Discussion

The electrolyte trajectories (Figure 3) in the ImNuT-cohort show a course comparable to what has previously been published (1, 42, 43). SGA infants are known to have a high risk of hypophosphatemia (3, 44), and the known triad of hypophosphatemia, hypokalemia and hypercalcemia (28) in the refeeding syndrome can be seen in the trajectories. The Kaplan–Meier plots show that pre-term infants who are SGA exhibited a markedly increased hazard of hypophosphatemia. Infants with GA ≥ 26 + 0 weeks showed a higher cumulative incidence of hypophosphatemia compared with those of lower GA; however, this difference did not reach statistical significance (*p* = 0.07). Our analysis of the ImNuT-cohort shows that SGA patients are overrepresented in the patients with higher GA. Thus, the contribution of SGA to hypophosphatemia risk may outweigh that of a low GA, potentially explaining the result.

Our study provides evidence for the potential of using ML models for reliable estimation of phosphate levels, both concurrently with blood gas analyses (T0) and ahead of time (predictively) (T12 and T24). The best performing models built on the main dataset achieved reproducible results across multiple ML algorithms (Table 4). Although predictive accuracy was moderate, the RMSE values suggest that the models may have potential clinical utility. In the internal validation scatter plots, model predictions of phosphate ≥ 2.0 mmol/L were never accompanied by measured values <1.5 mmol/L. This separation indicates that the models may have value as a screening tool, helping clinicians avoid unnecessary blood sampling when predicted phosphate levels are clearly within a safe range. The findings need to be reproduced in a prospective study before implementation. Classification results on the external validation dataset (Table 5) showed high sensitivity, but modest specificity and only moderate negative predictive value at the chosen threshold, suggesting it is better suited for identifying patients at risk of hypophosphatemia than for safely ruling it out.

External validation supported our findings, but RMSE was higher and R^2^ lower than for the best models in internal validation, indicating reduced performance. The external datasets were small (*n* = 43, 20 and 20), so RMSE and R^2^ should be interpreted with caution because the point estimates have substantial uncertainty. The range of phosphate values differed between the external datasets (0.18–2.5 mmol/L) and the ImNuT-dataset (0.61–3.05 mmol/L), which may have reduced performance when predicting values outside the range observed during model development. Notably, among the models we evaluated, the elastic nets showed the best performance in external validation. This pattern is consistent with the notion that models imposing a global linear structure may generalize more stably when extrapolating beyond the range of the development data (22). Furthermore, this may suggest that nonlinear interactions were limited in magnitude in this dataset given that the nonlinear models did not perform better than elastic net. The demographic characteristics (Table 2) between the ImNuT-cohort and the external validation cohort differ, which may explain some of the observed loss in predictive power.

Both protein and phosphate intakes were positively correlated with serum phosphate concentrations, with stronger correlations when intake was measured 24 h prior to the phosphate measurement than when measured concurrently. This lagged relationship is encouraging for the development of predictive models, as it underscores that nutritional management plays a role for changes in phosphate levels, and may offer an opportunity to intervene nutritionally to reduce the risk of hypophosphatemia. Across the three prediction tasks (T0, T12 and T24), six variables were consistently retained (calcium, potassium, SGA status, gestational age, birth weight *z*-score and daily weight *z*-score) indicating that these are among the most important predictors of phosphate concentrations in our models. Notably, several of these variables overlap with known risk factors for neonatal hypophosphatemia (3, 6).

ML-models can capture joint information of several variables, as demonstrated in our study (T0: 10 variables, T12: 8 variables and T24: 11 variables). The human brain cannot process all these variables in real time, which is why computer-assisted tools may benefit patients. By leveraging the models’ ability to integrate information from all available parameters, we can generate personalized forecasts of an infant's phosphate trajectories. In turn this can potentially improve the timeliness and precision of nutritional interventions aimed at preventing hypophosphatemia.

The principal strength of our study is the robustness of predictive performance across a variety of ML-algorithms and under three distinct data-pre-processing strategies: (1) Listwise deletion; (2) Listwise deletion and removal of outliers; and (3) Imputation of missing values. Outliers were identified using the MAD outlier test and results show that model performance was robust to outlier handling. A second advantage is the high-frequency sampling of phosphate values in the ImNuT-cohort during the first postnatal week. This abundance of response observations provides a solid foundation for supervised learning and supports the potential use of the models as bedside decision support tools.

### Study limitations

This single-center study was conducted under a standardized nutritional protocol, which may reduce some confounding related to nutritional management, but it also limits generalizability to settings with different practices. Our department has a strong focus on hypophosphatemia, with routine consideration of phosphate values and supplementation based on these results. Consequently, clinical management of hypophosphatemia, including phosphate supplementation in response to measured values, may have affected the results. Our external validation dataset is relatively small, and the range of phosphate values differed considerably from the range of values in the ImNuT-dataset. Another limitation concerns growth charts: we used the Niklasson growth chart for the ImNuT-dataset and Skjærven growth chart for the external validation dataset. Datasets using other growth standards may yield *z*-scores that are not directly comparable.

To our knowledge, this study is the first study to estimate and predict phosphate levels in preterm infants using ML. Building on these findings, prospective studies are needed to collect and label data specifically for this purpose, to refine the models and improve their generalizability. If successful, such models could become valuable tools for tailoring nutritional management to prevent hypophosphatemia, potentially reducing complications of preterm birth and subsequent neurocognitive impairment (4, 12). Furthermore, if phosphate values within the normal range can be reliably forecast, the need for unnecessary blood samples may be reduced, providing additional benefit to patients.

## Data Availability

Data presented in this manuscript is available from the corresponding author upon reasonable request, but is not publicly available due to GPDR restrictions. Requests to access these datasets should be directed to Asbjorn Westvik, aaswes@ous-hf.no.
