# Supplementary material for: Predicting serum phosphate levels in very preterm infants using machine learning
Source: Front Pediatr. 2026 Jul 15;14:1881474. doi: 10.3389/fped.2026.1881474 (PMC13415508; doi:10.3389/fped.2026.1881474)
Supplement: Supplementary file 1 [file Supplementaryfile1.docx]

***Supplementary Material***


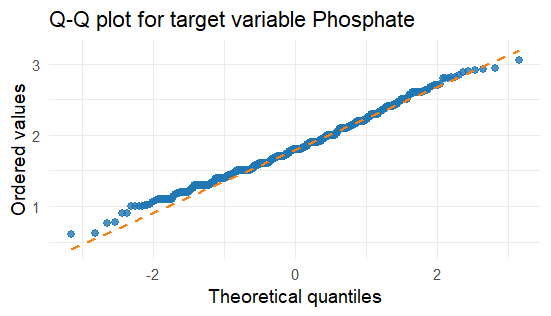
Supplementary Figure 1: Q-Q plot of Phosphate values

Supplementary Table 1: Model performance on T0, T12 and T24 across algorithms.

| **Internal** | **T0** |  | **T12** |  | **T24** |  |
| --- | --- | --- | --- | --- | --- | --- |
| **Validation** | **RMSE** | **R^2^** | **RMSE** | **R^2^** | **RMSE** | **R^2^** |
| **Elastic net SS** | 0.297 | 0.469 | 0.290 | 0.526 | 0.298 | 0.517 |
| **Elastic net YJ** | 0.295 | 0.475 | 0.289 | 0.530 | 0.297 | 0.519 |
| **GBR** | 0.299 | 0.460 | 0.293 | 0.517 | 0.296 | 0.521 |
| **KNN SS** | 0.311 | 0.417 | 0.306 | 0.473 | 0.297 | 0.519 |
| **KNN YJ** | 0.308 | 0.430 | 0.309 | 0.462 | 0.296 | 0.522 |
| **KR SS** | 0.426 | -0.091 | 0.305 | 0.479 | 0.433 | -0.022 |
| **KR YJ** | 0.384 | 0.112 | 0.296 | 0.508 | 0.323 | 0.430 |
| **RF** | 0.299 | 0.463 | 0.300 | 0.495 | 0.289 | 0.545 |
| **XGBoost** | 0.287 | 0.503 | 0.293 | 0.517 | 0.293 | 0.533 |
|  |  |  |  |  |  |  |
| **External** | **ext_T0** |  | **ext_T12** |  | **ext_T24** |  |
| **validation** | **RMSE** | **R^2^** | **RMSE** | **R^2^** | **RMSE** | **R^2^** |
| **Elastic net SS** | 0.308 | 0.562 | 0.360 | 0.581 | 0.584 | 0.047 |
| **Elastic net YJ** | 0.313 | 0.550 | 0.410 | 0.457 | 0.513 | 0.265 |
| **GBR** | 0.438 | 0.116 | 0.532 | 0.084 | 0.550 | 0.196 |
| **KNN SS** | 0.411 | 0.222 | 0.487 | 0.232 | 0.558 | 0.129 |
| **KNN YJ** | 0.392 | 0.292 | 0.503 | 0.181 | 0.537 | 0.196 |
| **KR SS** | 0.497 | -0.140 | 0.369 | 0.560 | 0.622 | -0.082 |
| **KR YJ** | 0.514 | -0.216 | 0.472 | 0.280 | 0.782 | -0.708 |
| **RF** | 0.421 | 0.183 | 0.617 | -0.234 | 0.568 | 0.100 |
| **XGBoost** | 0.484 | -0.078 | 0.618 | -0.238 | 0.666 | -0.240 |

Supplementary Table 2: Model performance on datasets without outliers and with imputed values

| **Removed missing**  **and outliers** | **T0** |  | **T12** |  | **T24** |  |
| --- | --- | --- | --- | --- | --- | --- |
|  | **RMSE** | **R^2^** | **RMSE** | **R^2^** | **RMSE** | **R^2^** |
| **Elastic net SS** | 0.289 | 0.457 | 0.288 | 0.531 | 0.298 | 0.445 |
| **Elastic net YJ** | 0.284 | 0.474 | 0.288 | 0.530 | 0.295 | 0.457 |
| **GBR** | 0.287 | 0.462 | 0.316 | 0.432 | 0.311 | 0.396 |
| **KNN SS** | 0.312 | 0.365 | 0.305 | 0.473 | 0.304 | 0.425 |
| **KNN YJ** | 0.309 | 0.378 | 0.308 | 0.464 | 0.305 | 0.418 |
| **KR SS** | 0.369 | 0.114 | 0.295 | 0.506 | 0.410 | -0.051 |
| **KR YJ** | 0.305 | 0.394 | 0.289 | 0.526 | 0.337 | 0.290 |
| **RF** | 0.295 | 0.431 | 0.298 | 0.498 | 0.299 | 0.443 |
| **XGBoost** | 0.279 | 0.492 | 0.307 | 0.466 | 0.306 | 0.417 |
|  |  |  |  |  |  |  |
| **Imputed missing**  **values** | **T0** |  | **T12** |  | **T24** |  |
|  | **RMSE** | **R^2^** | **RMSE** | **R^2^** | **RMSE** | **R^2^** |
| **Elastic net SS** | 0.292 | 0.499 | 0.299 | 0.497 | 0.345 | 0.398 |
| **Elastic net YJ** | 0.291 | 0.503 | 0.302 | 0.486 | 0.342 | 0.409 |
| **GBR** | 0.289 | 0.511 | 0.300 | 0.494 | 0.351 | 0.377 |
| **KNN SS** | 0.305 | 0.454 | 0.308 | 0.465 | 0.329 | 0.452 |
| **KNN YJ** | 0.305 | 0.454 | 0.305 | 0.477 | 0.330 | 0.449 |
| **KR SS** | 0.347 | 0.295 | 0.299 | 0.496 | 0.360 | 0.346 |
| **KR YJ** | 0.373 | 0.184 | 0.319 | 0.425 | 0.615 | -0.912 |
| **RF** | 0.294 | 0.492 | 0.300 | 0.493 | 0.317 | 0.492 |
| **XGBoost** | 0.280 | 0.541 | 0.290 | 0.525 | 0.322 | 0.475 |
